# Effect of molecular hydrogen treatment on Sepsis‐Associated encephalopathy in mice based on gut microbiota

**DOI:** 10.1111/cns.14043

**Published:** 2022-12-05

**Authors:** Qingqing Han, Yuanyuan Bai, Chunjing Zhou, Beibei Dong, Yingning Li, Ning Luo, Hongguang Chen, Yonghao Yu

**Affiliations:** ^1^ Department of Anaesthesiology Tianjin Medical University General Hospital, Tianjin Research Institute of Anaesthesiology Tianjin China; ^2^ Department of Anesthesiology Tianjin Baodi Hospital, Baodi Clinical College of Tianjin Medical University Tianjin China; ^3^ Department of Anaesthesiology Tianjin 4^th^ center hospital Tianjin China

**Keywords:** gut microbiota, hydrogen gas (H_2_), hydrogen‐rich water (HW), molecular hydrogen treatment, sepsis‐associated encephalopathy (SAE)

## Abstract

**Introduction:**

In our experiments, male wild‐type mice were randomly divided into four groups: the sham, SAE, SAE + 2% hydrogen gas inhalation (H_2_), and SAE + hydrogen‐rich water (HW) groups. The feces of the mice were collected for 16 S rDNA analysis 24 h after the models were established, and the serum and brain tissue of the mice were collected for nontargeted metabolomics analysis.

**Aim:**

Destruction of the intestinal microbiota is a risk factor for sepsis and subsequent organ dysfunction, and up to 70% of severely ill patients with sepsis exhibit varying degrees of sepsis‐associated encephalopathy (SAE). The pathogenesis of SAE remains unclear. We aimed to explore the changes in gut microbiota in SAE and the regulatory mechanism of molecular hydrogen.

**Results:**

Molecular hydrogen treatment significantly improved the functional outcome of SAE and downregulated inflammatory reactions in both the brain and the gut. In addition, molecular hydrogen treatment improved gut microbiota dysbiosis and partially amended metabolic disorder after SAE.

**Conclusions:**

Molecular hydrogen treatment promotes functional outcomes after SAE in mice, which may be attributable to increasing beneficial bacteria, repressing harmful bacteria, and metabolic disorder, and reducing inflammation.

## Graphical abstract

Molecular hydrogen treatment can significantly improve the gut microbiota and metabolites and reduce the inflammatory reaction and cognitive dysfunction after SAE. This study provides a basis for the pathogenesis and treatment of SAE.

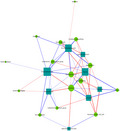

## 1 INTRODUCTION

Sepsis‐associated encephalopathy (SAE) is an important cause of the poor prognosis of patients with sepsis. It has been reported that SAE patients have a 20% higher mortality rate than septic patients without neurological symptoms.^1^ SAE pathogenesis and prevention strategies are crucial in emergency and critical care. Most of the current studies focus on the role of inflammatory mechanisms in sepsis.^2^, ^3^ The investigation of the intestinal flora and its metabolites is one of the most active research directions and frontiers in contemporary life sciences and medicine. In recent years, the international journals Nature, Science and Cell have published a series of papers emphasizing that an imbalance of the intestinal flora has an important influence on the pathogenesis of various human diseases, for example, metabolic, immunity‐related, gastrointestinal, and cognitive diseases.^4^, ^5^, ^6^ A new study has shown that the development of sepsis may be influenced by metabolic disorders of the intestinal flora and through the establishment of endogenous links between metabolites and other organs. Metabolic disorders of the intestinal flora mediate the multiorgan damage associated with sepsis.^7^

Hydrogen is the main gas in the gut, and it has been reported that 70% of gastrointestinal microorganisms genetically encode the ability to metabolize molecular hydrogen, suggesting that molecular hydrogen may affect the activities and community structure of intestinal microorganisms.^8^ It has been well recognized that microbial molecular hydrogen cycling plays an important role in gut microbial composition, metabolic homeostasis, and host health.^9^ Furthermore, hydrogen is an important mediator of the “microbial‐gut‐brain (MGB) axis”.^10^, ^11^, ^12^ Our previous studies showed that hydrogen strengthens the intestinal barrier and blood–brain barrier to provide protection against sepsis and SAE.^13^, ^14^, ^15^, ^16^ Interestingly, our recent study showed that drinking hydrogen‐rich water (HW) alters the composition and abundance of the gut microbiota.^17^ However, whether the protective effect of hydrogen on SAE is related to the metabolic activity of the intestinal flora and the specific underlying mechanism have not been determined. In this study, we studied the regulatory effect of molecular hydrogen treatment on the metabolic activity of the intestinal microbiota and the molecular pathways related to gut–brain axis material transport in SAE models by 16 S rDNA amplicon sequencing and nontargeted LC–MS/MS metabolomics analysis.

## 2 MATERIALS AND METHODS

### 2.1 Animal experiments

The Laboratory Animal Center of the Military Medical Science Academy in Beijing, China, provided us with male C57BL/6J mice aged 6–8 weeks, weighing 20–25 g each. The animals were housed at room temperature (20–22°C) and kept on a 12 h/12 h light/dark cycle, with food and water available at all times. The mice were randomly separated into four groups: the sham, SAE, SAE + 2% hydrogen gas inhalation (H_2_), and SAE + hydrogen‐rich water (HW) groups. The mice in the H_2_ group inhaled hydrogen for 1 h at 1 h and 6 h after the model was established. The hydrogen‐rich water was given to mice by gavage (0.01 ml/g), and the mice in all groups except the HW group received normal water. This study was approved by the Animal Care and Use Committee of Tianjin Medical University General Hospital. The mice in the above groups were sacrificed 24 h after the fear conditioning experiment, and then, the feces were collected for the analysis of microbial community diversity and untargeted metabonomic analysis. Moreover, we collected blood and brain tissue for untargeted metabonomic analysis. Brain tissue samples were also used to measure the expression levels of inflammatory cytokines (TNF‐α, IL‐6, and HMGB1) by ELISA kits.

### 2.2 2% H_2_ treatment

In accordance with our previous studies,^18^ the animals in the H_2_ treatment group were placed in a plastic box with an inlet and an outlet. H_2_ was administered by a TF‐1 gas flow meter (Yutaka Engineering Corp) and was mixed with air at a rate of 4 L/min. The H_2_ concentration in the box was continuously monitored by a detector (HY‐ALERTA Handheld Detector Model 500; H_2_ Scan) and was held at 2% throughout the treatment. Carbon dioxide was removed from the box with Baralyme.

### 2.3 Preparation of hydrogen‐rich water

The GCH‐300 high‐purity hydrogen generator (Tianjin Tongpu Analytical Instrument Technology Co., Ltd) was used to generate hydrogen gas from normal drinking water in a drinking bottle with an inlet and an outlet. Then, in order to prepare the HW (concentration 1200 ppb), 400 ml/min of hydrogen gas was blown into the water for 10 min. The gas flow rate was changed to 100 ml/min after 10 min, and the sample was blown into the drinking water to maintain a hydrogen molecule concentration of between 800 and 1000 ppb in the HW, and the exhaust gas was discharged into the exhaust gas recovery device through the outlet of the drinking bottle.

### 2.4 SAE model

SAE in mice was induced by caecal ligation and puncture (CLP), as described in a previous study, but with some modifications.^19^ Mice were placed on an operating table and anesthetized by isoflurane inhalation. The abdomen was prepped and disinfected with Iodophor. An approximately one‐centimeter‐long midline skin incision was made, and then, the caecum was isolated. Approximately 50% of the cecum was ligated with a silk suture, and the mesenteric artery was protected from destruction. Then, the ligated caecum was punctured twice with a 21‐gauge needle. The caecum was returned to the abdominal cavity after a small number of feces were squeezed out of the intestine. After the incision was closed with a 3–0 surgical suture, saline solution (1 ml) was injected into the mice subcutaneously, and lidocaine cream (Cat# H_2_0063466) was applied to alleviate their pain. The sham group only underwent laparotomy without caecal ligation or perforation.

### 2.5 Fear conditioning (FC)

According to a previous study,^20^ training was carried out 24 h prior to surgery. The mice were first familiarized with the environment for 120 s, and then given a 20 s 70 dB tone (conditional stimulus). At 25‐s intervals, a 0.70 mA electrical shock to the feet for 2 ms (unconditional stimulus) was given to the mice. After the shock was stopped, the next round of the experiment was carried out after an interval of 60 s, with a total of 6 repeats. The rigidity response was recorded and analyzed using an any‐maze video surveillance system. The evaluation experiment was performed at 6, 24, and 72 h postoperatively. Mice were placed in the same fear‐conditioning box (scenario related) and given the same conditional stimulus (conditional induction) as that administered during the training period. The freezing time of mice within 5 min was recorded, and the percentage of the conditionally induced freezing time was calculated (freezing time/total time × 100%) to evaluate the fear memory of the mice.

### 2.6 Sample collection and preparation

The mice were anesthetized by sevoflurane inhalation, and then, the thorax was cut open under aseptic conditions. We collected blood samples from the tip of the heart, and the supernatant was obtained by centrifugation after the samples were allowed to stand at room temperature for 1 h. Then, the mice were sacrificed. In the same aseptic conditions, the abdominal wall was cut open, the colon was pulled out and cut open, and stool samples were collected. Brain tissue samples were collected under aseptic conditions. Once collected, all specimens were stored in liquid nitrogen.

### 2.7 Microbial DNA extraction and sequencing

Total genomic DNA from the samples was extracted using the CTAB/SDS method. DNA concentration and purity were assessed on 1% agarose gels. PCR amplification of the 16 S V3–V4 region was performed using barcode‐specific primers (primer: 16 S V3–V4: 341F‐806R). In the next step, we separated the PCR products on a 2% agarose gel and purified them using the AxyPrep DNA Gel Extraction Kit (Axygen Biosciences, Union City). Then a sequencing library was produced through the NEB Next®Ultra™ DNA Illumina Library Preparation Kit (NEB) with an index code added according to the manufacturer's instructions. A Qubit@ 2.0 fluorometer (Thermo Scientific) and an Agilent Bioanalyzer 2100 system were used to assess the library quality. Finally, paired‐end reads of 250 bp/300 bp were generated after the library was sequenced on the Illumina MiSeq platform.

### 2.8 Analysis of 16 S rDNA‐sequencing data

FLASH was used to merge paired‐end reads from the original DNA fragments. According to the unique barcodes, each sample received paired‐end reads. An analysis of the sequence was conducted using the algorithms UPARSE‐OTUref and UPARSE‐OTU of the UPARSE software package. To analyze alpha (within the sample) and beta (among sample) diversities, internal Perl scripts were used. Ninety‐seven percent of sequences with similarity was assigned to the same OTU. A representative sequence was selected for each OTU to obtain the classification information with the RDP classifier. We rarefied the OTU table and calculated three indicators: Chao1, Simpson, and Shannon indices to calculate alpha diversity. The unweighted unifrac distance for Principal Coordinate Analysis (PCoA) performed by QIIME software was used to measure beta diversity.

### 2.9 LC/MS untargeted metabolomics analysis

Analysis of metabolites extracted after sample pretreatment was performed by a UHPLC (1290 Infinity LC, Agilent Technologies) with a quadrupole time‐of‐flight (AB Sciex TripleTOF 6600). The instrument was operated under the following conditions: chromatographic conditions—chromatographic column, ACQUITY UPLC BEH (2.1 mm × 100 mm, 1.7 μm); column temperature, 25°C; mobile phase, ammonium acetate, and hydroxide were mixed at 5 and 25 mM, respectively, in water (A) and acetonitrile (B); injection volume, 2 μl; flow rate, 0.5 ml/min; and mass spectrum condition—ion source, ESI. The ESI source conditions were set as follows: Ion Source Gas1 (Gas1) as 60, Ion Source Gas2 (Gas2) as 60, curtain gas (CUR) as 30, source temperature at 600°C, and IonSpray Voltage Floating (ISVF) ±5500 V. The mass spectrum signal acquisition of the samples included positive and negative ion scanning modes. Then, we preprocessed the raw data and analyzed it qualitatively and quantitatively using XCMS software.

### 2.10 ELISA

The mice brain tissue was lysed in RIPA/PMSF mixed liquids and centrifuged at 15000 rpm for 10 min at 4°C. Then the supernatants were analyzed by TNF‐α, IL‐6, and HMGB1 ELISA kits according to the manufacturer's instructions. The inflammatory cytokines concentrations were determined by comparison with the standard curve.

### 2.11 Statistical analysis

The statistical analysis of the data was performed by the Student's *t* test (SPSS 23.0) for analysis of variance. The differences in microbial communities were examined using ANOSIM, MRPPs, and Bray‐Curtis differential distance matrix analysis. LEfSe was applied for quantitative biomarker analysis in different groups. Results with *p* < 0.05 indicated statistical significance, and the significance test was a two‐tailed test.

## 3 RESULTS

### 3.1 Molecular hydrogen therapy improved the cognitive function and survival rate of clp mice

**FIGURE 1:**
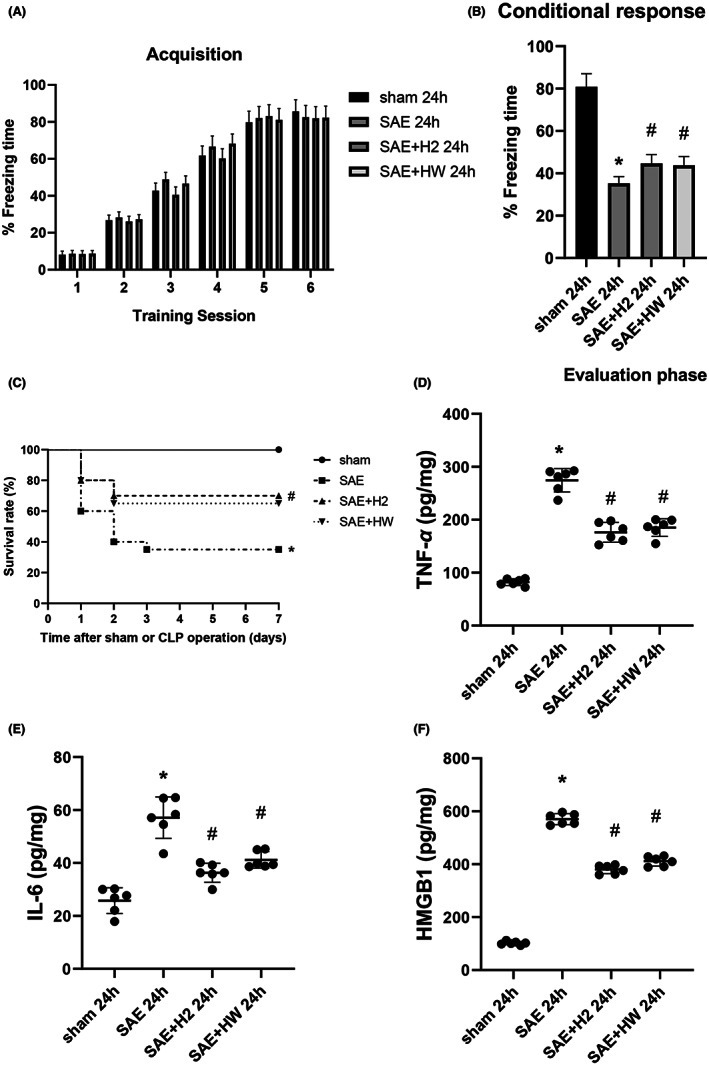
Effect of molecular hydrogen treatment on septic mice. The data plotted are the mean ± SD. (A) Training stage, (B) evaluation stage, (C) survival rate, (D) TNF‐α, (E) IL‐6, and (F) HMGB1 were measured by ELISA in each group (*n* = 6 mice per group). *p < 0.05 vs. sham group. #*p* < 0.05 vs. SAE group (*n* = 6 per group)

Before this experiment, we conducted 16 S rDNA amplification sequencing and behavioral experiments on the intestinal flora of mice at sham 6 h/24 h/72 h and SAE/6 h/24 h/72 h. We found that at 6 h, the composition and structure of intestinal flora in the model group mice had begun to change, but there was no brain dysfunction at this time. The intestinal microflora of SAE mice was most significantly changed at 24 h, whether β The diversity is also the relative abundance of species, and compared with 6 h and 72 h, the brain function of mice is damaged most obviously at 24 h (Figure S1). Therefore, we chose the time point of 24 h. Fear‐conditioning experiment was used to assess cognitive function at 24 h after CLP. Figure 1A,B shows that treatment with H_2_ or HW in CLP mice significantly reduced the degree of cognitive impairment 24 h after CLP. Furthermore, the survival rate of CLP mice was significantly increased after molecular hydrogen treatment, as shown in Figure 1C. Sepsis is a systemic inflammatory response, so we further verified the expression level of inflammatory factors. The results showed that the expression levels of TNF‐α, IL‐6, and HMGB1 in the SAE group and SAE with molecular hydrogen treatment groups (SAE + H_2_ group and SAE + HW group) were significantly higher than those observed in the sham group (*p* < 0.05). However, the expression of levels of TNF‐α IL‐6 and HMGB1 in brain homogenates in the SAE with molecular hydrogen treatment groups were lower than those observed in the SAE group (*p* < 0.05) (Figure 1D–F). These results suggested that molecular hydrogen therapy exerted a significant protective effect on mice with CLP.

### 3.2 Molecular hydrogen therapy partially restored gut microbiota disorders after SAE

**FIGURE 2:**
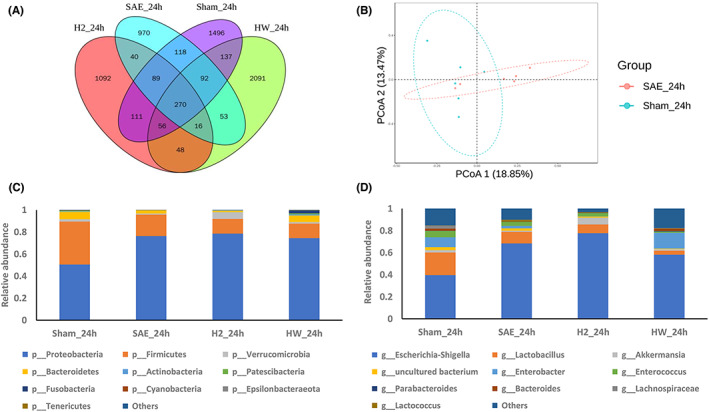
Effect of molecular hydrogen therapy on the gut microbiota of SAE mice. (A) The Venn diagram showed each group of unique and common OTUs. (B) PCoA shows differences between individuals or groups. The samples with high similarity of community structure tend to gather together, and the samples with large community differences tend to be far apart. Relative abundances of intestinal microbiota constituents at the (C) phylum and (D) genus levels in the sham, SAE, SAE + H_2_, and SAE + HW groups (*n* = 6 per group)

Feces were collected to perform 16 S rDNA sequencing to investigate the gut microbiota in the four groups. The common and unique OTUs among the different groups were shown by Venn diagrams (Figure 2A). After H_2_ treatment, there were 415 common OTUs and 2540 specific OTUs in the SAE group and H_2_ treatment group. After HW treatment, there were 431 common OTUs and 3549 unique OTUs in the SAE group and the HW group. The α diversity, including Chao1, Simpson, and Shannon indices, was used to analyze the diversity of microbial within the community. Chao1 index analysis showed that there was no distinct difference in microbial community richness among groups, while the Shannon and Simpson indices analyses also showed that these groups had similar community diversity (Table 1). In addition, the microbiome beta diversity manifested by principal coordinate analysis (PCoA) of samples was used to characterize the similarity or difference in community components between them. The separations in the scatterplot suggested that there were significant differences in community components between the SAE group and the sham group, indicating that sepsis obviously affected the composition of the gut microbiota (Figure 2B).

**TABLE 1:** Comparison of α diversity parameters between the sham group, SAE group, SAE + H_2_ group, and SAE + HW group.

Group	Chao 1	Shannon	Simpson
Sham	1312.09 ± 257.03	2.99 ± 0.53	0.69 ± 0.10
SAE	1111.17 ± 314.00	1.93 ± 1.25	0.45 ± 0.27
SAE + H_2_ group	1132.20 ± 207.10*	1.45 ± 0.76*	0.35 ± 0.20*
SAE + HW group	1577.85 ± 933.06*	2.46 ± 1.53*	0.50 ± 0.29*

*Note*: The values are presented as the mean ± SD.

* *p* > 0.05 vs. SAE group.

**FIGURE 3:**
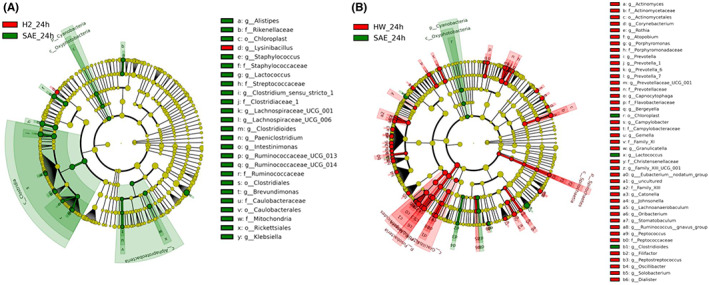
LEfSe analysis of gut microbiota. Cladogram of enriched taxa based on LEfSe determinations revealing significant differences in microbial communities between the (A) H_2_ and SAE groups and between the (B) HW and SAE groups (*n* = 6 per group). Bacterial taxa with an LDA score of >2 were selected as biomarker taxa (p, phylum level; c, class level; o, order level; f, family level; g, genus level).

Community structure component maps can show the community structure of each sample or group at different taxonomic levels. According to the species annotation results, the 10 species with the highest abundance at each classification level (phylum, class, order, family, genus, and species) were selected from each sample or group to generate column accumulation diagrams of relative abundance to visually display the species with a high relative abundance and their proportions at different classification levels. The composition of the fecal microbiota in mice treated with molecular hydrogen changed significantly at the phylum and genus levels (Figure 2C,D). The fecal microbiota of the SAE + H_2_ group showed a significant increase in the abundance of *Verrucomicrobia* and a decrease in the abundance of *Bacteroidetes* at the phylum level. At the genus level, the abundance of *Akkermansia* increased, whereas those of *Lactococcus* and *Lactobacillus* decreased. The abundances of *Bacteroidetes*, *Fusobacteria*, and *Actinobacteria* were increased at the phylum level in mice that drank HW. At the genus level, the abundances of *Akkermansia* and *Bacteroides* increased, whereas those of *Lactococcus*, *Lactobacillus*, *Escherichia‐Shigella*, and other harmful bacteria decreased. For LEfSe analysis, LDA was conducted on the samples on the basis of the taxonomic composition according to different grouping conditions, and microbial taxa with LDA values greater than 2 were identified; these microbes were considered to have been altered significantly (Figure 3A,B). At the genus level, inhalation of H_2_ increased the relative abundance of *Lysinibacillus* (*p* < 0.05). HW treatment increased the relative abundances of *Veillonella*, *Leptotrichia*, *Actinomyces*, and *Atopobium* in feces and decreased the relative abundance of *Klebsiella* and *Lactococcus* (*p* < 0.05).

### 3.3 Metabolomic profiles were altered significantly after molecular hydrogen treatment

**FIGURE 4:**
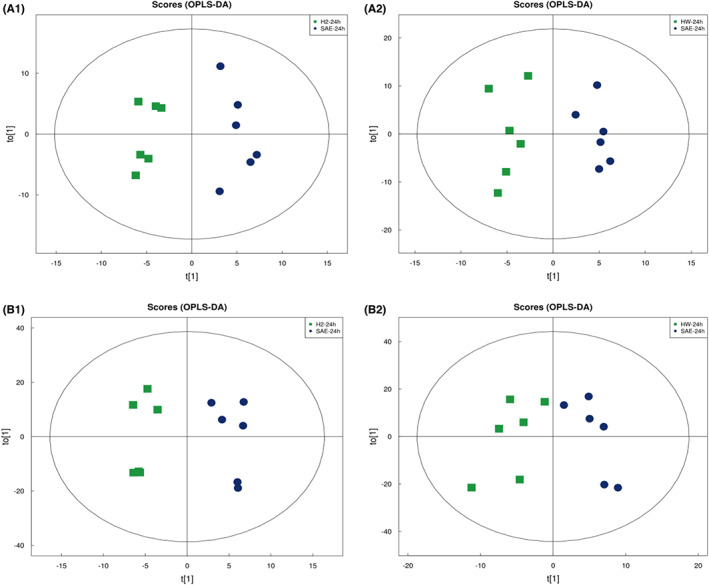
OPLS‐DA of serum and brain in the SAE group after H_2_ and HW treatments. (A1, B1): SAE + H_2_ group. (A2, B2): SAE + HW group. OPLS‐DA of the serum (A1, A2) and brain (B1, B2) show that the cluster of metabolites was significantly separated between the SAE group and postmolecular hydrogen treatment.

**FIGURE 5:**
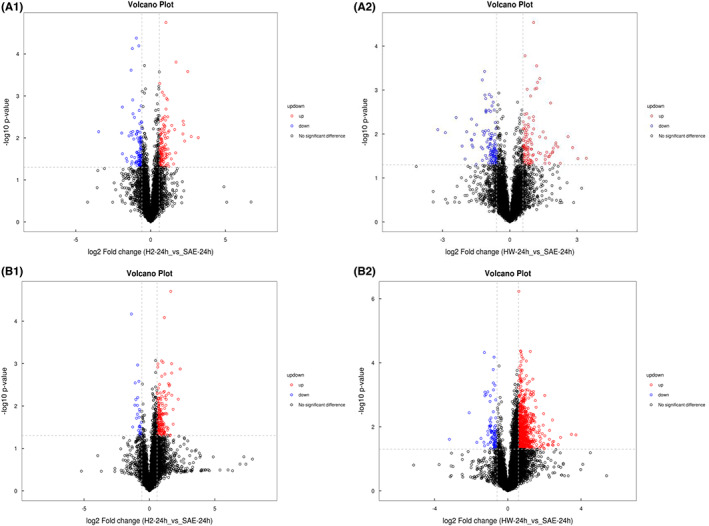
Volcano plot of serum and brain sample data from the SAE group after H_2_ and HW treatments. (A1, B1): SAE + H_2_ group. (A2, B2): SAE + HW group. Univariate statistical analysis of the volcano plot shows the metabolite changes in the serum (A1, A2) and brain (B1, B2) between the SAE group and postmolecular hydrogen treatment

To assess the difference in metabolites in SAE mice after H_2_ and HW treatments, we performed metabolomic analysis by LC–MS/MS and examined 24 serum samples and 24 brain tissue samples. We found that molecular hydrogen treatment shifted the metabolomic profiles of mice obviously. The OPLS‐DA score plot and volcano plot were used to screen for potential marker metabolites. Multivariate OPLS‐DA indicated that the point of metabolites was obviously separated between the SAE group and the postmolecular hydrogen treatment group, suggesting that the metabolism in the serum and brain was changed after molecular hydrogen treatment (Figure 4). Figure S2 shows that there is no overfitting phenomenon in the original model, and the model is robust. Univariate statistical analyses, such as volcano plot (Figure 5) analyses, also showed the changes in metabolites between the SAE and postmolecular hydrogen treatment groups.

**FIGURE 6:**
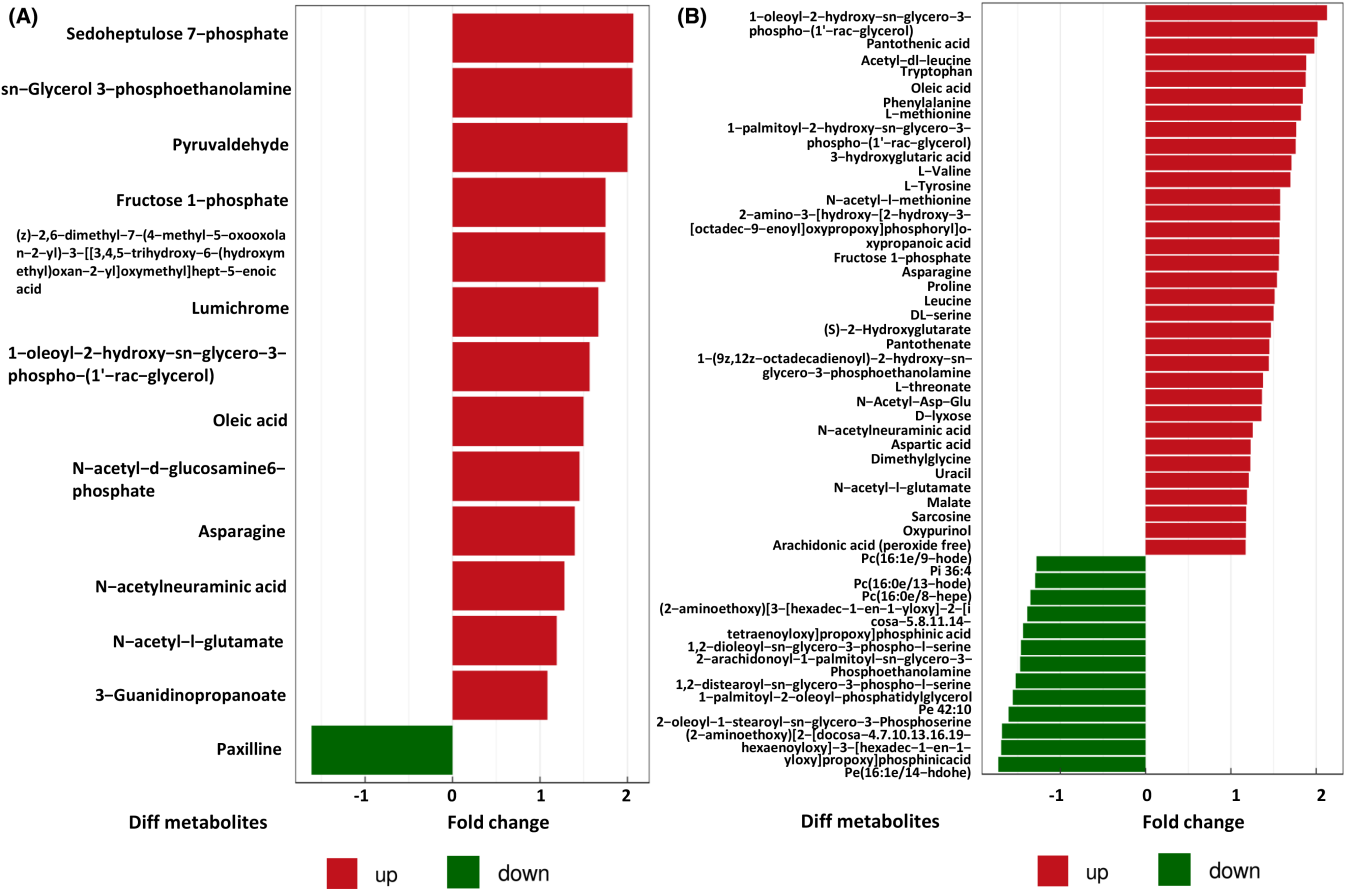
Negative ion mode significant difference brain metabolite analysis. (A) Significantly different metabolites. SAE + H_2_ group vs. SAE group (*p* < 0.05). (B) Significantly different metabolites. SAE + HW group vs. SAE group (*p* < 0.05)

**FIGURE 7:**
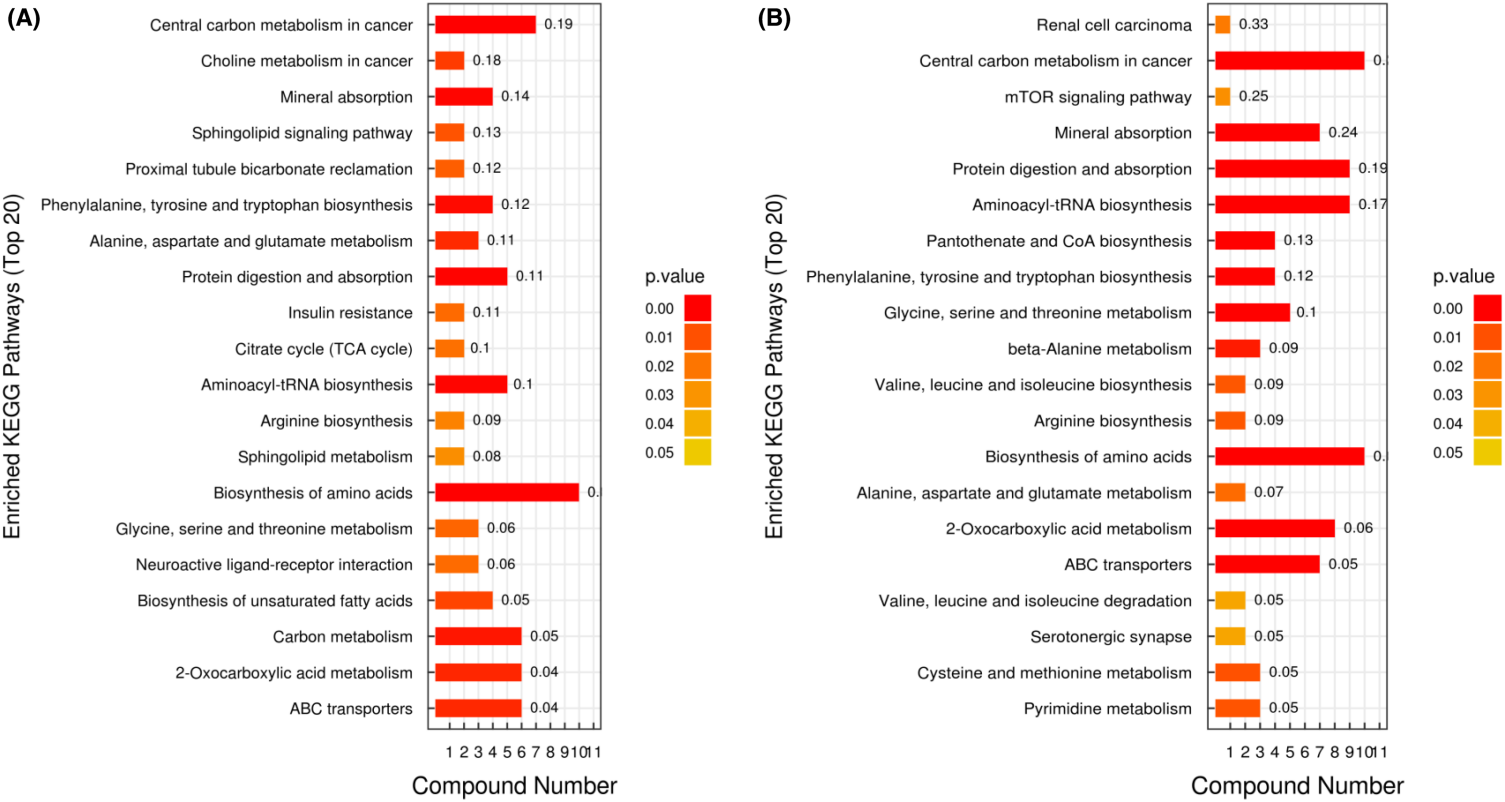
KEGG pathways enriched analysis. (A) KEGG pathways enriched in the SAE group compared with the sham group. (B) KEGG pathways enriched in the SAE + HW group compared with the SAE group

Fifty‐five potential brain metabolic biomarkers (VIP >1.0, *p* < 0. 05) were screened in SAE mice with molecular hydrogen treatment in total (Figure 6A,B). Among these, the relative production of 41 metabolites in the brain of mice significantly increased after molecular hydrogen treatment, whereas 14 metabolites obviously decreased. Most of these screened metabolites belonged to beneficial metabolites and showed an upward trend after molecular hydrogen treatment, such as Pantothenate, N‐acetylneuraminic acid, Sarcosine, Tryptophan, and DL‐serine. Moreover, KEGG pathway enrichment analysis was performed through the Fisher exact test. We found that a total of 20 pathways were highly altered. There were 11 common metabolic pathways compared with the metabolic pathways enriched by differential metabolites screened between the SAE group and the sham group. It was suggested that molecular hydrogen treatment may mainly affect SAE through these pathways, including ABC transporters, 2‐oxocarboxylic acid metabolism, alanine, aspartate and glutamate metabolism, biosynthesis of amino acids, arginine biosynthesis, glycine, serine and threonine metabolism, phenylalanine, tyrosine and tryptophan biosynthesis, aminoacyl−tRNA biosynthesis, protein digestion and absorption, mineral absorption, and central carbon metabolism in cancer (Figure 7A,B).

### 3.4 Changes in the expression level of the same metabolites in the serum and brain of SAE mice after molecular hydrogen treatment and the results of the joint analysis with gut microbiota

**FIGURE 8:**
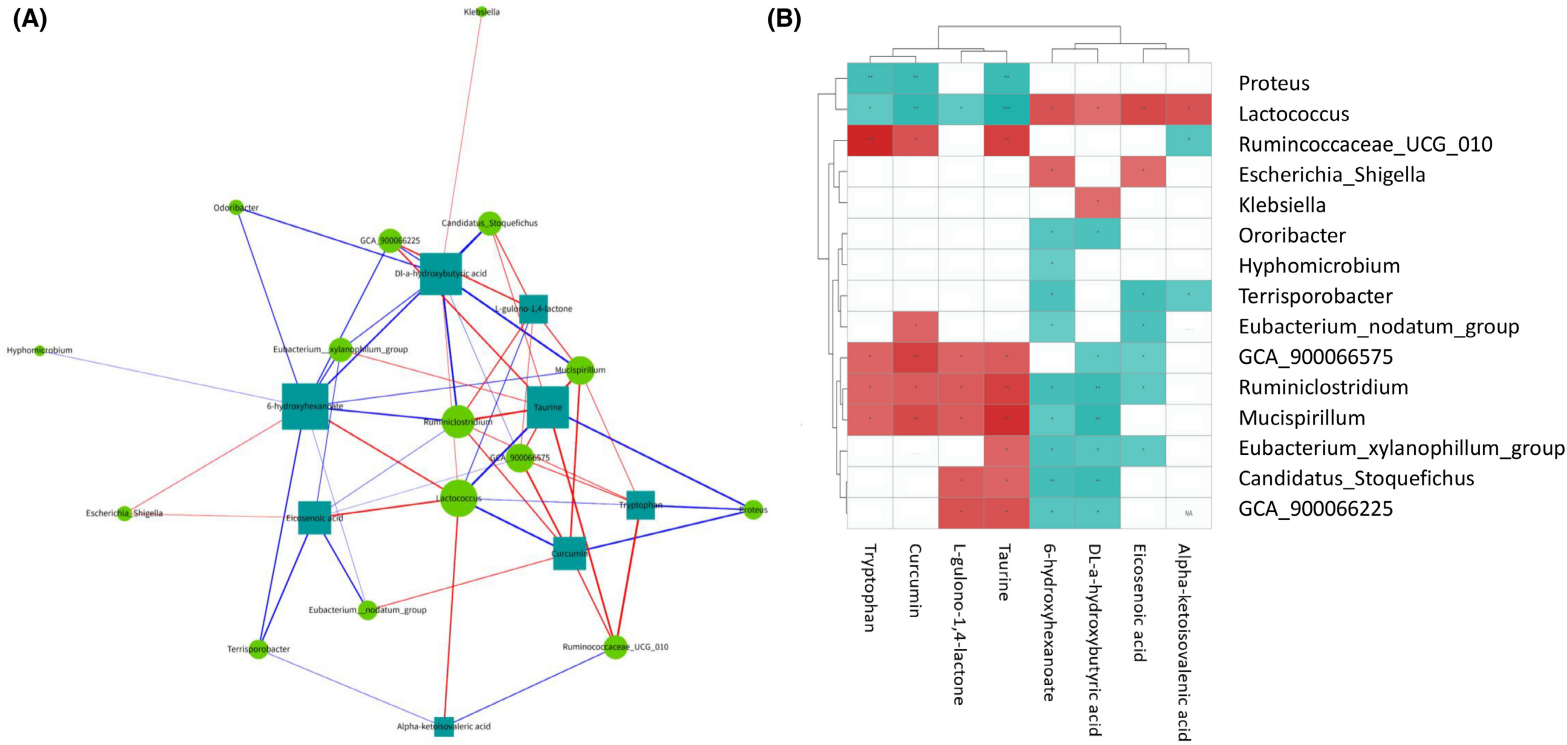
Spearman correlation analysis network diagram and Hierarchical clustering heatmap showing the associations between the metabolites and gut microbiota. (A) Spearman correlation analysis network diagram. The circle represents the gut microbiota, and the rectangle represents the metabolites. The blue line represents a negative correlation, the red line represents a positive correlation, and the thickness of the line is proportional to the absolute value of the correlation coefficient. The node size positively correlates with its degree, that is, the greater the degree, the larger the node size. (B) Hierarchical clustering heatmap of differentially expressed gut microbiota and metabolites.

In the past, sepsis was thought to occur mainly due to tissue and organ damage caused by an excessive inflammatory response. However, according to the latest studies, the pathogenesis of sepsis may be accompanied by common and specific metabolic changes in multiple tissues and organs (liver, lung, intestine, bone, and ilium muscle), suggesting that metabolic mechanisms may potentially underlie the pathogenesis of sepsis.^21^ Therefore, we screened the coaltered metabolites that showed significant differences in serum and brain tissue to explore the changes in metabolic levels in the SAE group and explored the changes in their expression levels in brain tissue after molecular hydrogen treatment (Table 2). Most of these metabolites are lipid and lipid‐like molecules, and the expression level in the brain of SAE mice after molecular hydrogen treatment has changed significantly. Then, correlation analysis between gut microbiota and metabolites was performed to evaluate the association between them. The Spearman correlation analysis network diagram (Figure 8A) and Hierarchical clustering heatmap (Figure 8B) provide a visualization of the relationship. For example, L‐gulono‐1,4‐lactone was positively correlated with *Candidatus_Stoquefichus* (*r* = 0.644), *GCA_900066225* (*r* = 0.693), *GCA_900066575* (*r* = 0.603), *Mucispirillum* (*r* = 0.651), and *Ruminiclostridium* (*r* = 0.652) but negatively correlated with *Lactococcus* (*r* = −0.622). Tryptophan level was positively correlated with the abundances of *GCA_900066575* (*r* = 0.624), *Mucispirillum* (*r* = 0.619), *Ruminiclostridium* (*r* = 0.621), and *Ruminococcaceae_UCG_010* (*r* = 0.877) but negatively correlated with the abundance of *Lactococcus* (*r* = −0.608) and *Proteus* (*r* = −0.72). These findings suggest that there were some common metabolic changes in the occurrence and development of SAE, and molecular hydrogen treatment may play an important protective role in SAE through the MGB axis.

**TABLE 2:** Changes of the coaltered metabolites (VIP >1.0, *p* < 0.05) in serum and brain tissue after H2 or HW treatment.

Metabolites	Fold change		SuperClass
	SAE + H_2_ vs SAE	SAE + HW vs SAE	
Taurine	1.07	1.09	Organic acids and derivatives
Dl‐a‐hydroxybutyric acid	1.10	1.21	Organic acids and derivatives
1,2‐distearoyl‐sn‐glycero‐3‐phospho‐l‐serine	0.90	0.66	Lipids and lipid‐like molecules
Lithosprmoside	1.85	2.17	Organicoxygen compounds
Pi (16:0 e/15‐hete)	1.44	1.64	Lipids and lipid‐like molecules
Pe (16:1 e/14‐hdohe)	0.88	0.58	Lipids and lipid‐like molecules
2‐methyl‐3‐hydroxybutyric acid	1.37	1.46	Lipids and lipid‐like molecules
Eicosenoic acid	1.27	1.73	Lipids and lipid‐like molecules
Pi 34:2	1.55	1.79	Lipids and lipid‐like molecules
Pc (16:0 e/8‐hepe)	0.89	0.72	Lipids and lipid‐like molecules
6‐hydroxyhexanoate	1.38	1.29	Organic acids and derivatives
Curcumin	2.16	1.89	Phenylpropanoids and polyketides
Tryptophan	1.39	1.88	Organoheterocyclic compounds
Alpha‐ketoisovaleric acid	2.14	1.55	Organic acids and derivatives
L‐gulono‐1,4‐lactone	0.68	0.73	Organoheterocyclic compounds

## 4 DISCUSSION

Our data showed that molecular hydrogen treatment improved cognitive function, suppressed inflammation in the brain, regulated metabolic disturbance, and inhibited the presence of harmful bacteria in mice after SAE.

Recent studies have shown that inflammatory microbiota, such as BAC303, EREC482, and LAB158, have changed significantly in stroke rats.^22^, ^23^ In the past 10 years, a large number of studies have shown that hydrogen has significant therapeutic effects on a variety of disease models as a new medical gas molecule.^24^ In this study, mice subjected to hydrogen inhalation and HW drinking were used to determine the mode of action of hydrogen therapy on the intestinal flora. There was no significant difference in α diversity in the gut microbiota of SAE mice treated with H_2_ or HW, but β diversity showed differences among the groups. We found that *Akkermansia* was significantly enriched at the genus level in the feces of the SAE group after treatment with hydrogen and HW by further identification of differential microorganisms. *Akkermansia muciniphila*, a second‐generation probiotic that is currently attracting much attention, is a typical intestinal microbe believed to confer longevity because of its effective alleviation of progeria.^25^, ^26^ Brahe et al found that low levels of Akk in the gut may lead to thinning of the mucosal layer, which leads to a weakened intestinal barrier and makes it easier for toxins in the gut to enter the body.^27^ Conversely, increasing the abundance of Akk can alleviate or even reverse metabolic disorders in mice.^28^ The abundance of *Proteobacteria* decreased significantly in the mice with HW. *Proteobacteria* is the main phylum of gram‐negative bacteria, including several pathogens, such as *Salmonella*, *Escherichia coli*, *Vibrio*, and *Helicobacter pylori*. Lipopolysaccharide (LPS) is a major component of the cell wall of gram‐negative bacteria and is one of the most effective inducers of the production of oxidative stress‐activated stimuli.^29^ Previous experiments have shown that the intestinal barrier becomes pathologically permeable under the effect of LPS.^29^, ^30^ Therefore, we speculate that the mechanism underlying the protective effects of molecular hydrogen therapy on SAE mainly involves increasing the abundance of beneficial flora and reducing the abundance of harmful flora to restore function in intestinal disorders.

The host interacts with its intestinal flora through metabolites.^31^ The intestinal flora is an important source of H_2_ production in vivo and plays a key role in host metabolism.^32^ To investigate changes in the metabolic function of the microbial community after H_2_ or HW treatment, we performed LC–MS/MS analysis. In this study, we found that after hydrogen and HW treatment, the levels of metabolites in the serum and brain tissue of the SAE group were significantly changed. We paid more attention to the beneficial metabolites with increased content after molecular hydrogen treatment. Sarcosine can be quickly converted into glycine in the body. As a structural amino acid, glycine plays an important role in human physiological processes and is the metabolic source of glutathione, creatine, purine, serine, and other essential components of living cells.^33^ Sarcosine plays an important role in schizophrenia, depression, and other mental diseases.^34^ N‐acetylneuraminic acid is the neurotransmitter of ganglioside. Some studies have shown that ganglioside (GM) is an essential substance for the regeneration and development of brain nerves, which can repair and promote the redevelopment of brain nerves.^35^, ^36^ These results suggest that molecular hydrogen therapy may improve the cognitive function of SAE mice mainly by improving metabolic disorders.

Akk can produce short‐chain fatty acids^37^, ^38^ and regulate lipid metabolism. A study has confirmed that some strains of AKK can produce vitamin B12, to promote the production of propionate.^39^ Hoyles et al. found that propionate suppressed pathways associated with nonspecific microbial infections via a CD14‐dependent mechanism, inhibited expression of LRP‐1, and protected the BBB from oxidative stress via NRF2 (NFE2L2) signaling.^40^ In addition, the latest research shows that short‐chain fatty acid sodium butyrate can combine GPR41 and GP43 to improve apoptosis and inflammation after stroke.^41^ Therefore, we speculate that molecular hydrogen treatment can increase the content of AKK, improve lipid metabolism, protect blood–brain barrier, and reduce brain function damage. But it is a pity that we did not find any metabolites significantly related to AKK in the joint analysis, which needs further exploration and verification.

Metabolites produced by gut microbes first enter the blood and then enter the brain, which is one of the ways in which gut microbes interact with the brain.^42^ To determine whether intestinal flora disorder affects the occurrence of SAE through this pathway and whether hydrogen also plays a protective role in SAE through this pathway, we screened the same differential metabolites in blood and brain before and after SAE and explored the changes of their expression levels after molecular hydrogen treatment. We found that the l‐gulono‐1,4‐lactone level decreased in the brain after molecular hydrogen treatment. L‐gulono‐1,4‐lactone can be transformed into ascorbic acid (AA). AA can provide beneficial effects for vascular health and alleviate oxidative stress and endothelial dysfunction by enhancing endothelial NO production.^43^ Therefore, a reduction in the l‐gulono‐1,4‐lactone level in blood is unfavorable. We speculate that the increase in the level of l‐gulono‐1,4‐lactone level, an organic heterocyclic compound, in brain tissue may have occurred due to damage to the blood–brain barrier during SAE,^44^, ^45^ resulting in the penetration and entry of macromolecules into the brain.^46^ The blood–brain barrier was repaired after molecular hydrogen treatment and made l‐gulono‐1,4‐lactone less permeable, which can also explain that its expression level in the blood increased but decreased in the brain. We noticed tryptophan among these same metabolites because it not only increased in blood and brain but also participated in multiple metabolic pathways of SAE regulated by molecular hydrogen treatment, whereas the other metabolites were not found to be associated with these metabolic pathways or had little association. Gut microbiota can regulate tryptophan metabolism.^47^ The results of 16 S rDNA and serum untargeted metabolomics association analysis also showed that tryptophan was related to a variety of gut microbiota. Tryptophan has multiple metabolic functions and plays an important role in immune homeostasis and intestinal barrier function.^48^ Tryptophan is the precursor of serotonin (5‐HT), an important neurotransmitter. When the content of 5‐HT in the brain decreases, it will show abnormal behavior, such as anxiety, nervous hallucinations, and insomnia. Appropriate supplementation of tryptophan can help relieve depressive symptoms.^49^ Therefore, we speculate that the mechanism of molecular hydrogen treatment to protect SAE may be to improve intestinal flora disorder, promote tryptophan biosynthesis and metabolism, and reduce the inflammatory response and brain dysfunction after sepsis.

Our research on microorganisms and metabolites only found a correlation between them. However, we have not identified how they are related and target SAE. Future research should verify the function of the candidate flora and explore the mechanism of its impact on the host to lay an experimental foundation for the discovery of a new mechanism for SAE treatment.

## AUTHOR CONTRIBUTIONS

Conceptualization: CHG and YYH; funding acquisition: YYH; methodology: HQQ and BYY; formal analysis and investigation: HQQ, LYN, and LN; writing—original draft: HQQ and ZCJ; writing—review and editing: DBB and YYH.

## FUNDING INFORMATION

This work was supported by the National Natural Science Foundation of China (grant no. 82072150) and the Tianjin Natural Science Foundation Project (grant no. 19JCYBJC25500).

## CONFLICT OF INTEREST

The authors declare that they have no competing interests.

## Supporting information


Figure S1.

Figure S2.

## Data Availability

The original contributions presented in the study are publiclyavailable. This data can be found here: https://www.ncbi.nlm.nih.gov/sra/PRJNA848371
