# Supplementary figures and images for: Effect of molecular hydrogen treatment on Sepsis‐Associated encephalopathy in mice based on gut microbiota
Source: CNS Neurosci Ther. 2022 Dec 5;29(2):633–45. doi: 10.1111/cns.14043 (PMC9873526; doi:10.1111/cns.14043)

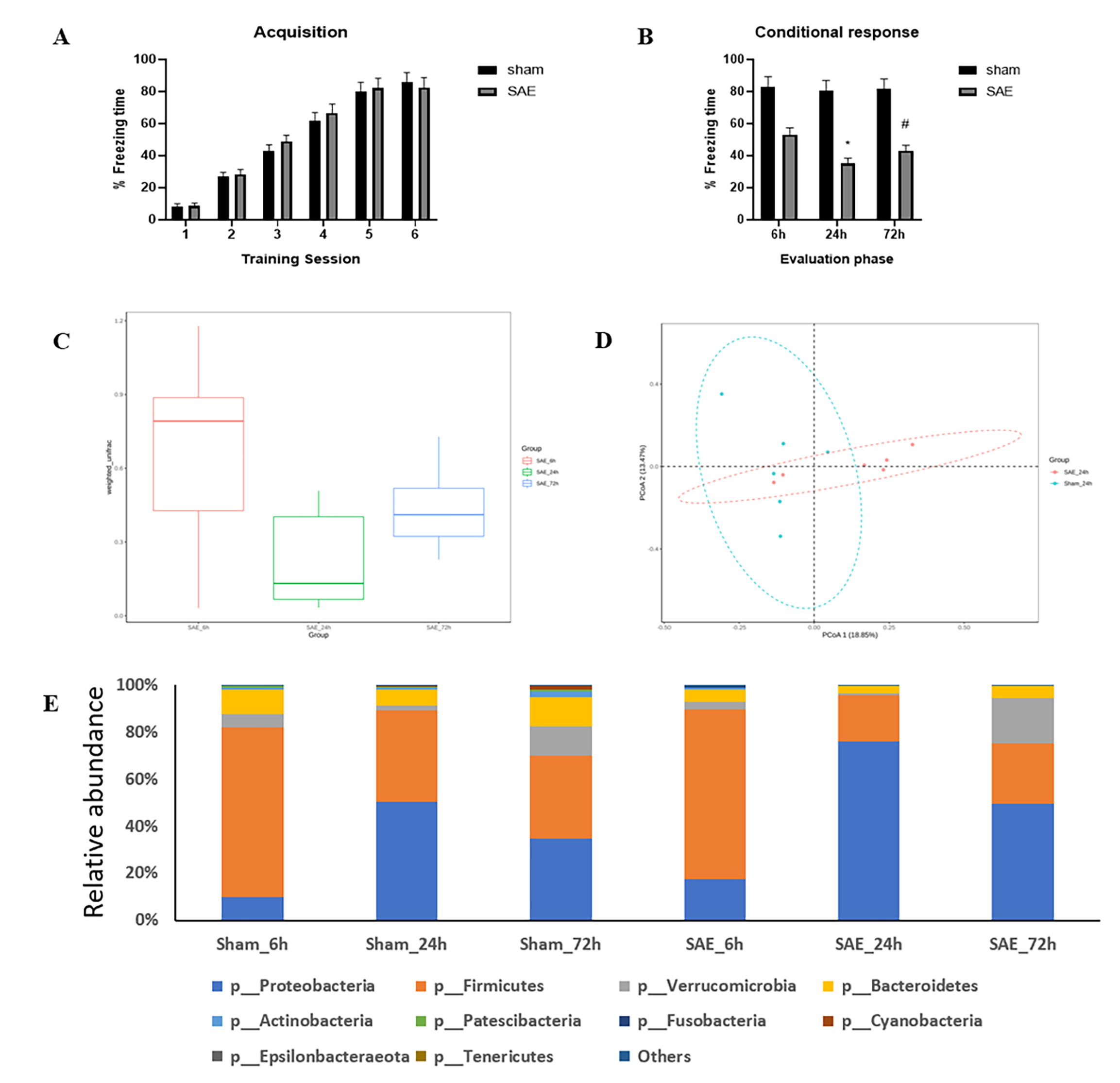

Supplement: Supplementary file 1 — Figure S1. [file CNS-29-633-s002.tif]

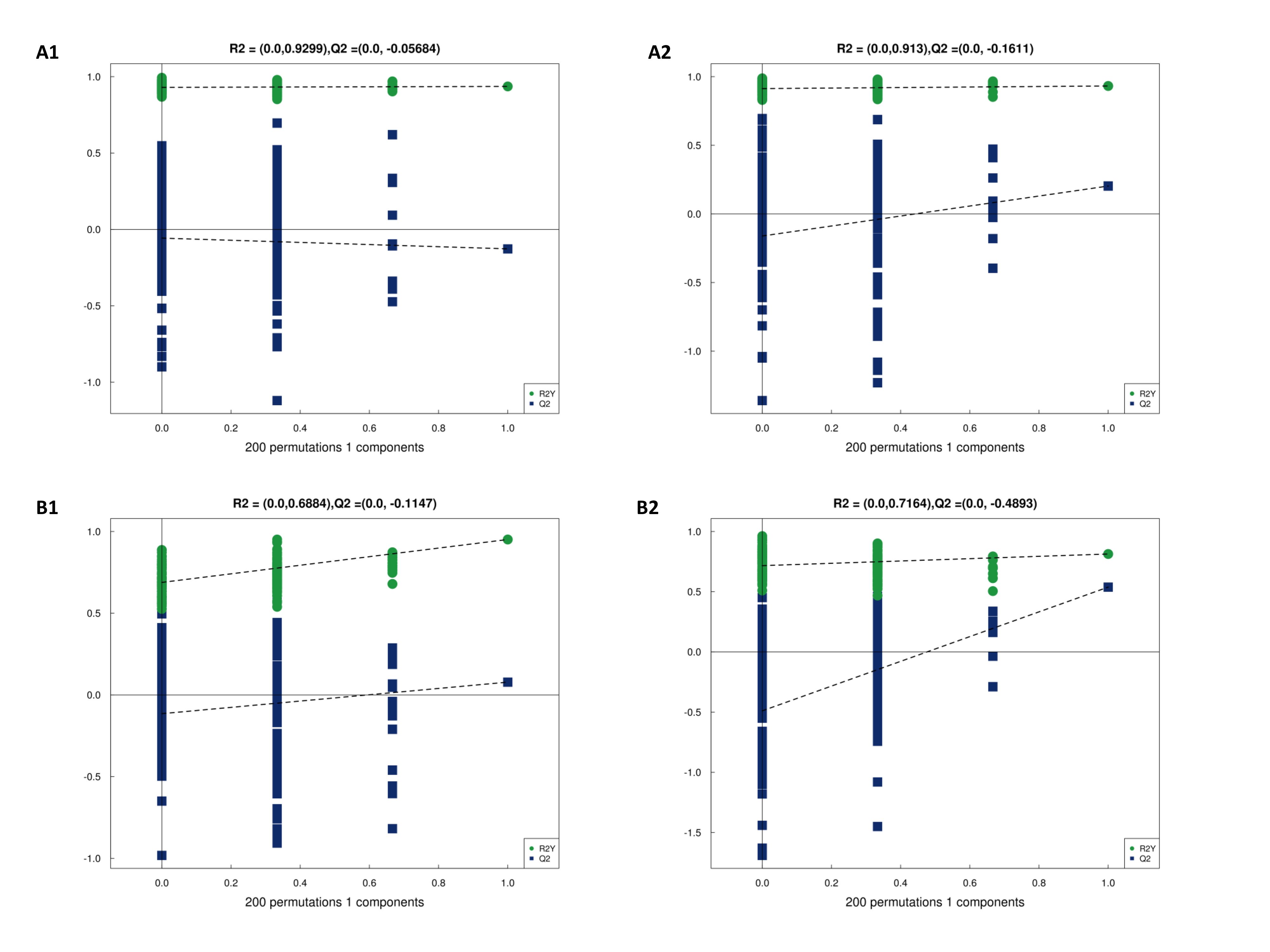

Supplement: Supplementary file 2 — Figure S2. [file CNS-29-633-s001.tif]
